# Profiles of Cough and Associated Risk Factors in Nonhospitalized Individuals With SARS-CoV-2 Omicron Variant Infection: Cross-Sectional Online Survey in China

**DOI:** 10.2196/47453

**Published:** 2024-02-05

**Authors:** Tingting Xu, Yuehan Chen, Wenzhi Zhan, Kian Fan Chung, Zhongmin Qiu, Kewu Huang, Ruchong Chen, Jiaxing Xie, Gang Wang, Min Zhang, Xuefen Wang, Hongmei Yao, Xiuqing Liao, Yunhui Zhang, Guojun Zhang, Wei Zhang, Dejun Sun, Jia Zhu, Shujuan Jiang, Juntao Feng, Jianping Zhao, Gengyun Sun, Huaqiong Huang, Jianyong Zhang, Lingwei Wang, Feng Wu, Suyun Li, Pusheng Xu, Chunhua Chi, Ping Chen, Mei Jiang, Wen He, Lianrong Huang, Wei Luo, Shiyue Li, Nanshan Zhong, Kefang Lai, China Cough Coalition

**Affiliations:** 1 State Key Laboratory of Respiratory Disease National Clinical Research Center for Respiratory Disease, Guangzhou Institute of Respiratory Health The First Affiliated Hospital of Guangzhou Medical University Guangzhou China; 2 Department of Respiratory Medicine Royal Brompton and Harefield Hospital London United Kingdom; 3 Experimental Studies Unit National Heart & Lung Institute Imperial College London London United Kingdom; 4 Department of Pulmonary and Critical Care Medicine School of Medicine Tongji Hospital, Tongji University Shanghai China; 5 Department of Pulmonary and Critical Care Medicine Beijing Chao-Yang Hospital Capital Medical University Beijing China; 6 West China School of Medicine West China Hospital of Sichuan University Chengdu China; 7 Department of Respiratory and Critical Care Medicine Shanghai General Hospital Shanghai JiaoTong University School of Medicine Shanghai China; 8 Department of Respiratory Medicine The First Affliated Hospital, Zhejiang University School of Medicine Hangzhou China; 9 Department of Respiratory Medicine Guizhou Provincial People’s Hospital Guiyang, Guizhou China; 10 Department of Respiratory Medicine Fuling Center Hospital of Chongqing Chongqing China; 11 The First People's Hospital of Yunnan Province Kunming China; 12 Department of Respiratory and Critical Care Medicine The First Affiliated Hospital of Zhengzhou University Zhengzhou China; 13 Department of Respiratory and Critical Care Medicine The First Affiliated Hospital of Nanchang University Nanchang China; 14 Department of Pulmonary and Critical Care Medicine the Inner Mongolia Autonomous Region People's Hospital Hohhot China; 15 Department of Respiratory Medicine Jiangsu Province Hospital of Chinese Medicine, Affiliated Hospital of Nanjing University of Chinese Medicine Nanjing China; 16 Department of Pulmonary Medicine Shandong Provincial Hospital Affiliated to Shandong University Jinan China; 17 Department of Respiratory Medicine Xiangya Hospital, Central South University Changsha China; 18 Department of Respiratory Medicine Tongji Hospital, Tongji Medical College of Hua Zhong University of Science and Technology Wuhan China; 19 Department of Respiratory Medicine The First Affiliated Hospital of Medical University of Anhui Hefei China; 20 Key Laboratory of Respiratory Disease of Zhejiang Province Department of Respiratory and Critical Care Medicine Second Affiliated Hospital of Zhejiang University School of Medicine Hangzhou China; 21 The Second Department of Pulmonary and Critical Care Medicine Affiliated Hospital of Zunyi Medical University Zunyi China; 22 Shenzhen People's Hospital Shenzhen Institute of Respiratory Diseases Shenzhen China; 23 Department of Pulmonary and Critical Care Medicine Huizhou Third People's Hospital, Guangzhou Medical University Huizhou China; 24 The First Affiliated Hospital of Henan University of Chinese Medicine Zhengzhou China; 25 The Second Affiliated Hospital of Guangzhou Medical University Guangzhou China; 26 Department of Respiratory and Critical Care Medicine Peking University First Hospital Beijing China; 27 General Hospital of Northern Theater Command Shenyang China; 28 See Acknowledgments

**Keywords:** COVID-19, Omicron variant, nonhospitalized, cough

## Abstract

**Background:**

Cough is a common symptom during and after COVID-19 infection; however, few studies have described the cough profiles of COVID-19.

**Objective:**

The aim of this study was to investigate the prevalence, severity, and associated risk factors of severe and persistent cough in individuals with COVID-19 during the latest wave of the Omicron variant in China.

**Methods:**

In this nationwide cross-sectional study, we collected information of the characteristics of cough from individuals with infection of the SARS-CoV-2 Omicron variant using an online questionnaire sent between December 31, 2022, and January 11, 2023.

**Results:**

There were 11,718 (n=7978, 68.1% female) nonhospitalized responders, with a median age of 37 (IQR 30-47) years who responded at a median of 16 (IQR 12-20) days from infection onset to the time of the survey. Cough was the most common symptom, occurring in 91.7% of participants, followed by fever, fatigue, and nasal congestion (68.8%-87.4%). The median cough visual analog scale (VAS) score was 70 (IQR 50-80) mm. Being female (odds ratio [OR] 1.31, 95% CI 1.20-1.43), having a COVID-19 vaccination history (OR 1.71, 95% CI 1.37-2.12), current smoking (OR 0.48, 95% CI 0.41-0.58), chronic cough (OR 2.04, 95% CI 1.69-2.45), coronary heart disease (OR 1.71, 95% CI 1.17-2.52), asthma (OR 1.22, 95% CI 1.02-1.46), and gastroesophageal reflux disease (GERD) (OR 1.21, 95% CI 1.01-1.45) were independent factors for severe cough (VAS>70, 37.4%). Among all respondents, 35.0% indicated having a productive cough, which was associated with risk factors of being female (OR 1.44, 95% CI 1.31-1.57), having asthma (OR 1.84, 95% CI 1.52-2.22), chronic cough (OR 1.44, 95% CI 1.19-1.74), and GERD (OR 1.22, 95% CI 1.01-1.47). Persistent cough (>3 weeks) occurred in 13.0% of individuals, which was associated with the risk factors of having diabetes (OR 2.24, 95% CI 1.30-3.85), asthma (OR 1.70, 95% CI 1.11-2.62), and chronic cough (OR 1.97, 95% CI 1.32-2.94).

**Conclusions:**

Cough is the most common symptom in nonhospitalized individuals with Omicron SARS-CoV-2 variant infection. Being female, having asthma, chronic cough, GERD, coronary heart disease, diabetes, and a COVID-19 vaccination history emerged as independent factors associated with severe cough, productive cough, and persistent cough.

## Introduction

The SARS-CoV-2 virus has been spreading globally since 2019, causing over 664 million infections and 6.7 million deaths as of January 14, 2023 [1]. The epidemiology and clinical characteristics of COVID-19 have been widely studied; however, SARS-CoV-2 has continued to mutate from the dominant Alpha to Delta variants and now the Omicron variant and subvariants, and the clinical characteristics of COVID-19 also change according to the particular virus variant [2-4]. The Omicron variant has been the dominant variant in the fourth wave of the global COVID‐19 pandemic having infected an estimated 300 million people worldwide as of January 2023. Since December 2022, infection with the Omicron variant has been spreading rapidly across China.

Omicron is recognized as being highly contagious with a high capability for immune escape [5,6]. Over half of patients are reported to have at least one symptom during the infection period, including fever, cough, sore throat, fatigue, dyspnea, headache, and body pain [2,4,7]. Among these symptoms, cough is one of the most common [8,9]. Experiencing a chronic cough is associated with a significant decrease in quality of life, with symptoms such as incontinence, chest pain, headache, and poor sleep [10]. Studies in the United Kingdom [3] and the United States [4] have shown that the prevalence of acute cough during Omicron infection ranges from approximately 67.4% to 78%. A study conducted in a mobile cabin hospital in Shanghai demonstrated that cough was present in 57.5% of 1139 patients [7]. In addition, nearly 2.4%-7.2% of patients still experience cough as a symptom 1-2 months after Omicron infection is resolved [8,9]. However, few studies have described the specific cough features such as its severity, pattern, and risk factors.

We hypothesized that certain risk factors might be related with severe cough, productive cough, and persistent cough after Omicron infection. Additionally, there has been no nationwide survey concerning the clinical characteristics of Omicron infection in China to date. Therefore, we investigated the prevalence, severity, and associated risk factors for cough in nonhospitalized individuals with Omicron infection during the latest COVID-19 wave (2022-2023) in China.

## Methods

### Study Design and Participants

A nationwide survey was conducted online by China Cough Coalition [11], which is an organization dedicated to promoting the regular diagnosis, management, and research on cough in China, and has been assessing the cough characteristics of the Omicron pandemic. From December 31, 2022, to January 11, 2023, we recruited individuals aged ≥18 years with positive nucleic acid or antigen detection of SARS-CoV-2 or highly suspected symptoms of COVID-19; individuals with mental illness or other conditions that might hinder their ability to complete the survey were excluded. Only nonhospitalized individuals were included in the study.

Data were collected using Wenjuanxing [12], an online questionnaire survey platform in China. Participants were asked to fill out the questionnaire by using their smartphone, and each device was restricted to submitting the questionnaire results only once. The questionnaires were mainly distributed by physicians of China Cough Coalition and the survey link was also available for the public. At the top of the questionnaire, the purpose and contents of the survey were briefly introduced and participants were required to read the informed consent and confirm that they voluntarily agreed to join this survey. Participants had full right to quit the study at any time during the survey.

### Ethics Considerations

This study was approved by the Ethics Review Committees of the First Affiliated Hospital of Guangzhou Medical University (ES-2023-012-01). Although verbal consent was provided as described above, the requirement for written informed consent was waived by the institutional ethics board of the hospital. No financial compensation was offered to the subjects for their participation. All data were collected anonymously and promised to be used only in this study.

### Questionnaire Items

A standardized and structured questionnaire was designed for this study. Prior to the distribution of the final version of the questionnaire, it was first tested in a pilot study (data not shown). The questionnaire consisted of 21 items, divided into the following six aspects (see Multimedia Appendix 1): (1) baseline data (age, sex, smoking history, vaccination history for COVID-19, and comorbidities); (2) detection of SARS-CoV-2 (date of the positive result of SARS-CoV-2 antigen/nucleic acid testing); (3) general and severe symptoms, including date of onset of COVID-19 symptoms (cough, fever, fatigue, myalgia/arthralgia, nasal congestion, headache/dizziness, sore throat, runny nose, hyposmia/hypogeusia, chest tightness, diarrhea, postnasal drip, and conjunctivitis; see Multimedia Appendix 1 for the definition of “severe” symptoms); (4) cough characteristics, including the date of cough onset, cough pattern, type of sputum, duration of cough, timing of cough, and cough severity compared with previous common cold experience; (5) results of chest computed tomography/chest X-ray according to radiology reports if available; and (6) medication, including antipyretics, antibiotics, antiviral drugs, antitussives, nasal drugs, and traditional Chinese medicine. Cough severity was evaluated using a visual analog scale (VAS), with a range of 0-100 mm, where 0 indicates no cough and 100 indicates the worst cough imaginable [13]. Severe cough was defined as VAS>70. Persistent cough was defined as a cough duration >3 weeks and chronic cough was defined as a cough duration >8 weeks.

### Statistical Analysis

Categorical variables are presented as frequency (percentage), while continuous variables are expressed as mean (SD) or median (IQR). Statistical comparisons between groups were performed with unpaired Student *t* tests for normally distributed data, Mann-Whitney *U* tests for skewed data, and *χ*^2^ tests or Fisher exact tests for proportional data. Multivariable logistic regression was performed to detect the risk factors for persistent cough adjusted by age, sex, and duration from infection to the survey, assessed according to estimates of adjusted odds ratios, 95% CIs, and *P* values [14]. The final model selection was determined according to the backward selection of all factors based on the Akaike information criterion. Statistical analysis was performed using R software (v 4.1.2).

## Results

### Demographic and General Information of Participants

A total of 12,227 questionnaires were recorded in 31 provinces across China. The number of questionnaires in different provinces is shown in Multimedia Appendix 2. We excluded 78 individuals with incorrect information and 431 hospitalized individuals. Therefore, data were analyzed from a total of 11,718 nonhospitalized individuals, including clinic attendees (15.4%) and individuals not diagnosed or treated in hospitals (84.6%).

The demographic characteristics of the individuals are shown in Table 1. Among the 11,718 individuals with a median age of 37.0 (IQR 30.0-47.0) years, 7978 (68.1%) were female. The median duration from Omicron infection to the survey was 16.0 (IQR 12.0-20.0) days. The majority of individuals were not current smokers and had no comorbidities. The most frequent comorbidities were chronic rhinitis/sinusitis, followed by hypertension, chronic urticaria/allergic dermatitis, and asthma.

Among the total sample, the most common symptoms were cough, followed by fever, fatigue, and nasal congestion. The most common severe symptoms included severe bone pain/myalgia, high fever, and severe nasal congestion (Table 2).

**Table 1:** Baseline characteristics of nonhospitalized participants with Omicron variant infection (N=11,718).

Characteristics			Value
Female, n (%)			7978 (68.1)
Age (years), median (IQR)			37.0 (30.0-47.0)
Positive nucleic acid/antigen test, n (%)			9950 (84.9)
Duration from infection to survey (days), median (IQR)			16.0 (12.0-20.0)
Clinic visits, n (%)			1804 (15.4)
Vaccination^a^, n (%)			11,259 (96.1)
**Last vaccination^b^, n (%)**			
	≤6 months	1707 (15.2)	
	>6 months	9552 (84.8)	
**Medication, n (%)**			
	Antipyretics	8262 (70.5)	
	Traditional Chinese medicine	5127 (43.8)	
	Antitussive	2987 (25.5)	
	Antibiotics	2280 (19.5)	
	Antiviral drugs	356 (3.0)	
	Nasal drugs	229 (2.0)	
	None	1007 (8.6)	
**Smoking status, n (%)**			
	Current smoker	906 (7.7)	
	Nonsmoker	10,812 (92.3)	
**Comorbidities, n (%)**			
	COPD^c^	136 (1.2)	
	Asthma	546 (4.7)	
	Chronic cough	495 (4.2)	
	Interstitial lung disease	35 (0.3)	
	Chronic rhinitis/sinusitis	1838 (15.7)	
	Hypertension	843 (7.2)	
	Diabetes	314 (2.7)	
	Coronary heart disease	121 (1.0)	
	Cerebrovascular disease	78 (0.7)	
	GERD^d^	521 (4.4)	
	Malignant tumor	191 (1.6)	
	Chronic kidney disease	59 (0.5)	
	Chronic urticaria/allergic dermatitis	644 (5.5)	
	None of the above diseases	7578 (64.7)	

^a^Inactivated vaccine for SARS-CoV-2.

^b^Proportions were calculated only for patients with a COVID-19 vaccination history.

^c^COPD: chronic obstructive pulmonary disease.

^d^GERD: gastroesophageal reflux disease.

**Table 2:** Prevalence of general and severe symptoms in nonhospitalized individuals with Omicron variant infection (N=11,718).

Symptoms		Participants, n (%)
**General symptoms**		
	Cough	10,741 (91.7)
	Fever	10,243 (87.4)
	Fatigue	9407 (80.3)
	Nasal congestion	8058 (68.8)
	Myalgia/arthralgia	8033 (68.6)
	Headache/dizziness	7740 (66.1)
	Sore throat	7576 (64.7)
	Runny nose	6901 (58.9)
	Hyposmia/hypogeusia	5403 (46.1)
	Chest tightness	4019 (34.3)
	Diarrhea	2628 (22.4)
	Postnasal drip	1887 (16.1)
	Conjunctivitis	571 (4.9)
	Others	712 (6.1)
**Severe symptoms**		
	Severe bone pain/myalgia	4858 (41.5)
	High fever (≥39.1℃)	4558 (38.9)
	Severe nasal congestion	4129 (35.2)
	Severe sore throat	3851 (32.9)
	Severe headache	3366 (28.7)
	Dyspnea	1867 (15.9)
	None of the above	2438 (20.8)

### Cough Characteristics

Among the 10,741 respondents with cough, 44.0% (n=4724) presented with cough during both daytime and nighttime and 36.8% (n=3956) only had the cough during the daytime (Table 3). In terms of the cough pattern, 35.0% (n=3760) of individuals presented with a productive cough; white thick sputum and yellow purulent sputum were common.

Coughs were commonly initiated within 3 days, as reported by 6580 (61.3%) of the 10,741 individuals with cough. At entry to the survey, 38.2% (n=4103) of individuals still had cough, while 61.8% (n=6638) of individuals reported that their cough was relieved. Among the 2209 individuals with a duration from infection to the survey over 3 weeks, the cough was relieved within 1 week in 23.9% (n=528) of individuals, lasted for 1-3 weeks in 52.6% (n=1162) of individuals, developed into a subacute cough (3-8 weeks) in 12.7% (n=281) of individuals, and developed into a chronic cough (>8 weeks) in 0.3% (n=8) of individuals.

**Table 3:** Characteristics of cough in individuals with cough (N=10,741).

Characteristics			Value
Female, n (%)			7466 (69.5)
Age (years), median (IQR)			37.0 (30.0-46.0)
Duration from infection to survey (days), median (IQR)			16.0 (12.0-20.0)
Cough onset time (days), median (IQR)			3.0 (2.0-4.0)
Cough duration (weeks), median (IQR)			10.0 (7.0-14.0)
**Cough duration (weeks), n (%)**			
	<1	3975 (37.0)	
	1-3	6424 (59.8)	
	3-8	331 (3.1)	
	>8	11 (0.1)	
Cough VAS^a^, median (IQR)			70 (50-80)
**Cough compared to previous common cold, n (%)**			
	Less severe	639 (5.9)	
	Similar	2525 (23.5)	
	More severe	7577 (70.5)	
**Cough timing, n (%)**			
	Both daytime and nighttime	4724 (44.0)	
	Mainly daytime	3956 (36.8)	
	Mainly evening (before falling asleep)	1377 (12.8)	
	Mainly nighttime (after falling asleep)	684 (6.4)	
**Cough pattern, n (%)**			
	Nonproductive	6981 (65.0)	
	Productive	3760 (35.0)	
**Property of sputum^b^, n (%)**			
	White and thick viscous	809 (21.5)	
	Yellow and then white	737 (19.6)	
	White with a little yellow	644 (17.1)	
	Yellow and purulent	613 (16.3)	
	White and then yellow	536 (14.3)	
	White and watery	286 (7.6)	
	Blood-stained	135 (3.6)	

^a^VAS: visual analog scale.

^b^Proportions were calculated among patients with productive cough.

### Factors Associated With Severe, Productive, and Persistent Cough

With a cut-off VAS score of 70, 4388 (37.4%) of the total 11,718 respondents were categorized in the severe cough group and 7330 (62.6%) were categorized in the nonsevere group. Compared with those in the nonsevere group, the group with severe cough had a higher proportion of female participants (*P*<.001), a younger age (*P*<.001), fewer current smokers (*P*<.001), more individuals who had been vaccinated for COVID-19 (*P*<.001), and higher proportions of individuals with gastroesophageal reflux disease (GERD) (*P*=.03) and chronic cough (*P*<.001) as comorbidities (Table 4).

With respect to the different patterns of cough, more female respondents presented with a productive cough than male respondents (*P*<.001). In addition, the group with a productive cough included higher proportions of individuals with COPD (*P*=.01), asthma (*P*<.001), chronic cough (*P*<.001), interstitial lung disease (*P*=.03), chronic rhinitis/sinusitis (*P*=.003), and GERD (*P*=.008) as comorbidities (Table 5).

The group with a persistent cough included higher proportions of individuals with their most recent vaccination injected longer than 6 months before (*P*=.03) and higher proportions of comorbidities, including asthma (*P*=.005), chronic cough (*P*<.001), chronic rhinitis/sinusitis (*P*=.02), and diabetes (*P*=.002) compared to the group with acute cough (Table 6).

**Figure 1:**
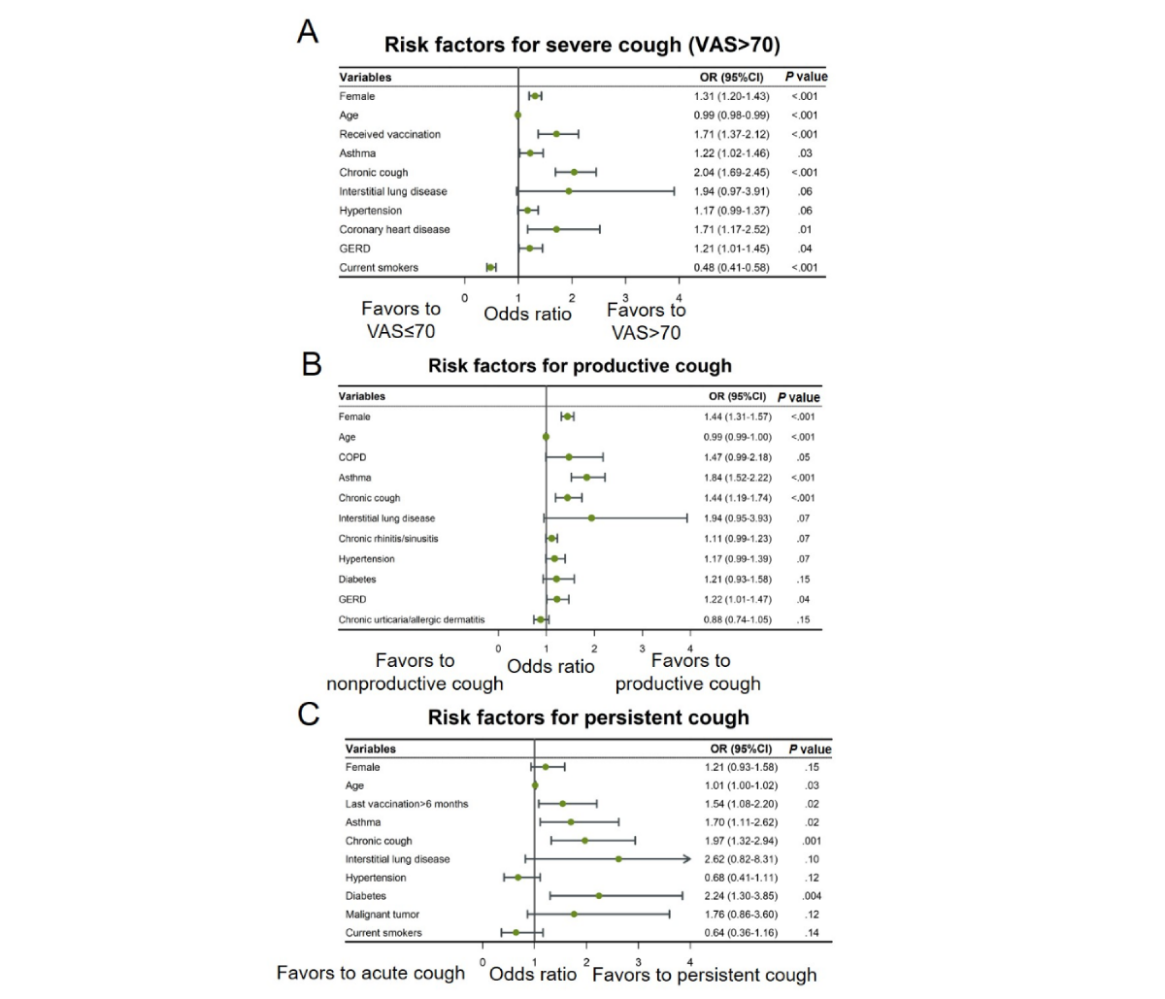
Forest plot of risk factors for severe cough (VAS>70), productive cough, and persistent cough. (A) Adjusted odds ratios (ORs) of factors for severe cough (VAS>70) versus nonsevere cough (VAS≤70). (B) Adjusted ORs of risk factors for productive cough versus nonproductive cough. (C) Adjusted ORs of risk factors for persistent cough versus acute cough. Adjusted by age, sex, and duration from infection to survey. COPD: chronic obstructive pulmonary disease; GERD: gastroesophageal reflux disease; VAS: visual analog scale.

Multivariate analysis showed that being female; being younger; having received vaccination for COVID-19; having asthma, chronic cough, coronary heart disease, or GERD as comorbidities; and being a nonsmoker were independent factors associated with more severe cough (Figure 1A). Being female, of younger age, and having asthma, chronic cough, and GERD as comorbidities were independent factors for productive cough (Figure 1B). The most recent vaccination more than 6 months ago, and having asthma, chronic cough, and diabetes were independent factors for persistent cough (Figure 1C).

**Table 4:** Comparison of characteristics between individuals with severe and nonsevere cough.^a^

Characteristics		Total (N=11,718)	Nonsevere cough (n=7330)	Severe cough (n=4388)	*P* value	
Female, n (%)		7978 (68.1)	4753 (64.8)	3225 (73.5)	<*.*001	
Age (years), median (IQR)		37.0 (30.0-47.0)	38.0 (30.0-48.0)	36.0 (29.0-45.0)	<*.*001	
Duration from infection to survey (days), median (IQR)		16.0 (12.0-20.0)	16.0 (12.0-20.0)	16.0 (12.0-20.0)	*.*06	
Vaccination^b^, n (%)		11259 (96.1)	6991 (95.4)	4268 (97.3)	<*.*001	
**Last vaccination^c^, n (%)**						*.*60
	≤6 months	1707 (15.2)	1070 (15.3)	637 (14.9)		
	>6 months	9552 (84.8)	5921 (84.7)	3631 (85.1)		
**Comorbidities, n (%)**						
	COPD^d^	136 (1.2)	96 (1.3)	40 (0.9)	*.*06	
	Asthma	546 (4.7)	325 (4.4)	221 (5.0)	*.*15	
	Chronic cough	495 (4.2)	231 (3.2)	264 (6.0)	<*.*001	
	Interstitial lung disease	35 (0.3)	16 (0.2)	19 (0.4)	*.*06	
	Chronic rhinitis/sinusitis	1838 (15.7)	1155 (15.8)	683 (15.6)	*.*80	
	Hypertension	843 (7.2)	549 (7.5)	294 (6.7)	*.*12	
	Diabetes	314 (2.7)	209 (2.9)	105 (2.4)	*.*15	
	Coronary heart disease	121 (1.0)	70 (1.0)	51 (1.2)	*.*33	
	Cerebrovascular disease	78 (0.7)	47 (0.6)	31 (0.7)	*.*76	
	GERD^e^	521 (4.4)	302 (4.1)	219 (5.0)	*.*03	
	Malignant tumor	191 (1.6)	119 (1.6)	72 (1.6)	>.99	
	Chronic kidney disease	59 (0.5)	42 (0.6)	17 (0.4)	*.*22	
	Chronic urticaria/allergic dermatitis	644 (5.5)	389 (5.3)	255 (5.8)	*.*26	
	None of the above diseases	7578 (64.7)	4760 (64.9)	2818 (64.2)	*.*44	
**Smoking status, n (%)**						<*.*001
	Current smoker	906 (7.7)	718 (9.8)	188 (4.3)		
	Nonsmoker	10812 (92.3)	6612 (90.2)	4200 (95.7)		

^a^Severe cough was defined as a visual analog scale score>70; the score for individuals without cough was set to 0.

^b^Received an inactivated vaccine for SARS-CoV-2.

^c^Proportions were calculated only for patients with a COVID-19 vaccination history.

^d^COPD: chronic obstructive pulmonary disease.

^e^GERD: gastroesophageal reflux disease.

**Table 5:** Comparison of characteristics between individuals with or without productive cough.

Characteristics		Total (N=10,741)	Nonproductive cough (n=6981)	Productive cough (n=3760)	*P* value	
Female, n (%)		7466 (69.5)	4676 (67.0)	2790 (74.2)	<.001	
Age (years), median (IQR)		37.0 (30.0-46.0)	37.0 (30.0-47.0)	36.0 (29.0-45.0)	<.001	
Duration from infection to survey (days), median (IQR)		16.0 (12.0-20.0)	16.0 (12.0-20.0)	16.0 (12.0-20.0)	.53	
Vaccination^a^, n (%)		10361 (96.5)	6739 (96.5)	3622 (96.3)	.62	
**Last vaccination^b^, n (%)**						.39
	≤6 months	1555 (15.0)	996 (14.8)	559 (15.4)		
	>6 months	8806 (85.0)	5743 (85.2)	3063 (84.6)		
**Comorbidities, n (%)**						
	COPD^c^	112 (1.0)	60 (0.9)	52 (1.4)	.01	
	Asthma	496 (4.6)	250 (3.6)	246 (6.5)	<.001	
	Chronic cough	470 (4.4)	263 (3.8)	207 (5.5)	<.001	
	Interstitial lung disease	33 (0.3)	15 (0.2)	18 (0.5)	.03	
	Chronic rhinitis/sinusitis	1729 (16.1)	1070 (15.3)	659 (17.5)	.003	
	Hypertension	750 (7.0)	484 (6.9)	266 (7.1)	.82	
	Diabetes	272 (2.5)	171 (2.4)	101 (2.7)	.50	
	Coronary heart disease	105 (1.0)	65 (0.9)	40 (1.1)	.57	
	Cerebrovascular disease	67 (0.6)	43 (0.6)	24 (0.6)	.99	
	GERD^d^	494 (4.6)	293 (4.2)	201 (5.3)	.008	
	Malignant tumor	178 (1.7)	113 (1.6)	65 (1.7)	.73	
	Chronic kidney disease	50 (0.5)	31 (0.4)	19 (0.5)	.77	
	Chronic urticaria/allergic dermatitis	609 (5.7)	396 (5.7)	213 (5.7)	>.99	
	None of the above diseases	6936 (64.6)	4610 (66)	2326 (61.9)	<.001	
**Smoking status, n (%)**						.13
	Current smoker	679 (6.3)	460 (6.6)	219 (5.8)		
	Nonsmoker	10,062 (93.7)	6521 (93.4)	3541 (94.2)		

^a^Recieved an inactivated SARS-CoV-2 vaccine.

^b^Proportions were calculated only among patients with a COVID-19 vaccination history.

^c^COPD: chronic obstructive pulmonary disease.

^d^GERD: gastroesophageal reflux disease.

**Table 6:** Comparison of characteristics between individuals with persistent cough and acute cough.

Characteristics		Total (N=10,741)	Acute cough^a^ (n=10,399)	Persistent cough (n=342)	*P* value	
Female, n (%)		7466 (69.5)	7213 (69.4)	253 (74.0)	.08	
Age (years), median (IQR)		37.0 (30.0-46.0)	37.0 (30.0-46.0)	38.5 (32.0-48.0)	.003	
Duration from infection to survey (days), median (IQR)		16.0 (12.0-20.0)	16.0 (12.0-20.0)	26.0 (23.0-29.0)	<.001	
Vaccination^b^, n (%)		10361 (96.5)	10029 (96.4)	332 (97.1)	.63	
**Last vaccination^c^, n (%)**						.03
	≤6 months	1555 (15.0)	1520 (15.2)	35 (10.5)		
	>6 months	8806 (85.0)	8509 (84.8)	297 (89.5)		
**Comorbidities, n (%)**						
	COPD^d^	112 (1.0)	108 (1.0)	4 (1.2)	.78	
	Asthma	496 (4.6)	469 (4.5)	27 (7.9)	.005	
	Chronic cough	470 (4.4)	438 (4.2)	32 (9.4)	<.001	
	Interstitial lung disease	1729 (16.1)	1668 (16.0)	61 (17.8)	.42	
	Chronic rhinitis/sinusitis	33 (0.3)	29 (0.3)	4 (1.2)	.02	
	Hypertension	750 (7.0)	728 (7.0)	22 (6.4)	.77	
	Diabetes	272 (2.5)	254 (2.4)	18 (5.3)	.002	
	Coronary heart disease	105 (1.0)	100 (1.0)	5 (1.5)	.39	
	Cerebrovascular disease	67 (0.6)	62 (0.6)	5 (1.5)	.06	
	GERD^e^	494 (4.6)	476 (4.6)	18 (5.3)	.64	
	Malignant tumor	178 (1.7)	168 (1.6)	10 (2.9)	.10	
	Chronic kidney disease	50 (0.5)	49 (0.5)	1 (0.3)	>.99	
	Chronic urticaria/allergic dermatitis	609 (5.7)	587 (5.6)	22 (6.4)	.62	
	None of the above diseases	6936 (64.6)	6743 (64.8)	193 (56.4)	.002	
**Smoking status, n (%)**						.07
	Current smoker	679 (6.3)	666 (6.4)	13 (3.8)		
	Nonsmoker	10062 (93.7)	9733 (93.6)	329 (96.2)		

^a^Acute cough: duration of cough ≤7 days and without cough at the time of this survey.

^b^Received an inactivated SARS-CoV-2 vaccine.

^d^Proportions were calculated only among patients with a COVID-19 vaccination history.

^d^COPD: chronic obstructive pulmonary disease.

^e^GERD: gastroesophageal reflux disease.

## Discussion

To the best of our knowledge, this is the first study with a large sample size to investigate the characteristics of cough and associated factors in nonhospitalized individuals infected with the SARS-CoV-2 Omicron variant in China. In this study, the prevalence of cough was 92.6%, followed by fever, fatigue, myalgia/arthralgia, nasal congestion, headache/dizziness, sore throat, and runny nose. Productive cough (35.0%) with white thick sputum or yellow purulent sputum was common, which developed into a persistent cough in a subset of the surveyed individuals. Female sex, being a current smoker, and having comorbidities (such as asthma, chronic cough, GERD, and/or diabetes) were factors associated with severe, productive, and persistent cough.

Early reports showed that the prevalence of cough during COVID-19 infection ranged from 44% to 72.5% [15], with a higher prevalence associated with Omicron variant infection than with Delta variant infection [4], although one survey from the United Kingdom [3] indicated that the prevalence of cough was similar between infections with the Omicron and Delta variants. In this study, the prevalence of cough was 92.6%, which was higher than that reported in previous studies [2-4,7], especially those based on data collected prior to emergence of the Omicron variant [16-18], suggesting that the prevalence of cough might be higher with the Omicron variant than with previous variants. In addition to cough, other symptoms such as fever, fatigue, myalgia, nasal congestion, and sore throat were also common, and over 60% of individuals reported having severe symptoms in our study.

With 15 amino acid substitutions in the receptor-binding domain, Omicron has been identified as being highly transmissible and possessing a remarkable capability of immune escape [19]. It has been reported that Omicron caused less damage to the lungs compared with Delta [20] and was more likely to target the upper airways [21]. The vagal sensory nerves play essential roles in regulating cough, which terminate primarily in the larynx, trachea, carina, and large intrapulmonary bronchi [22]. This may explain the greater prevalence of cough among patients with Omicron variant infection. The other possibility for the higher proportion of cough and other symptoms identified in this study might be related to the reduced herd immunity against SARS-CoV-2. After the outbreak of COVID-19 in early 2020, a very strict prevention and control policy was implemented in China; thus, most people have not been exposed to COVID-19 in recent years [23]. In addition, the latest pandemic outbreak in China occurred during the winter, when viruses multiply more rapidly and spread more quickly on the one hand and the immune response of the airway is decreased on the other hand.

Previous studies have reported that compared to female patients, male patients with COVID-19 are at a higher risk of hospitalization, with worse outcomes and a higher mortality rate, independent of age [24]. In our study, all participants had mild-to-moderate disease and had not been hospitalized for COVID-19. We found that the female participants reported a higher proportion of cough and more severe cough. There was also a female predominance in the group reporting a chronic cough, which could be related to the heightened cough sensitivity among female patients [25]. This female preponderance in the chronic cough population and enhanced cough sensitivity may be explained by sex-related differences in the central processing of cough sensation [26]. However, further studies are needed to determine whether female patients with COVID-19 also present with increased cough sensitivity.

In China, 25.6% of adults (50.7% of men and 1.9% of women) are smokers [27]. The high proportion of nonsmoking female respondents in our study may explain why the proportion of smokers in our data was lower than that of the general population. Smoking has been reported as a risk factor for severe symptoms and/or progression of COVID-19 [28,29], and the prevalence of chronic cough and sputum was reported to be greater among current smokers [30]. Interestingly, smokers tended to report a nonsevere cough in the current survey. Many cigarette smokers usually have a chronic cough, but they scarcely seek medical attention unless a change in the pattern or intensity of their cough is noticed [31]. Cough reflex sensitivity and sensitivity to airway irritation were reported to be significantly decreased in current smokers compared to those of a similar population of nonsmokers [32,33]. Smoking was also found to be accompanied by increased activation in brain regions known to be involved in both cough sensory processing and cough suppression. Therefore, the smoking-induced sensitization of central cough neural circuits could be offset by concurrently enhanced central suppression [32].

Cough is a frequent and common complaint of patients with respiratory diseases such as asthma, chronic obstructive pulmonary disease, and interstitial lung disease. Nevertheless, cough could also be a sole or predominant symptom in extrapulmonary disorders, particularly GERD [34,35]. Most of the available evidence indicates that patients with respiratory diseases or cardiovascular diseases have the greatest odds of cough [9,36,37]. We also found an association of comorbidities, including asthma, chronic cough, coronary heart disease, GERD, and/or diabetes, with severe cough, persistent cough, and productive cough. Many studies have suggested a significant increase in the severity and mortality of COVID-19 in people with diabetes [38]. Self-reported chronic cough/phlegm was more common in patients with diabetes compared to that of the general population [39]. Gliptin, a dipeptidyl peptidase-4 inhibitor used to treat diabetes mellitus, can cause cough as a side effect, which may be attributed to the persistent cough of COVID-19 [40]. In addition, diabetes, as an important risk factor, was found to influence the clinical severity of concomitant viral, bacterial, and fungal infections [41]. Therefore, attention should be paid to the possibility of coinfection or secondary bacterial infection in cases of persistent cough among patients with diabetes with COVID-19.

Approximately one-third of the individuals surveyed in this study reported having a productive cough, indicating the hypersecretion of mucus. In addition, the group with productive cough had a low proportion of individuals with respiratory comorbidities, which could not fully explain the excessive phlegm production. Excessive mucus production and accumulation is a common response to respiratory viral infections such as respiratory syncytial virus, rhinovirus, influenza virus, and SARS virus [42]. Recently, Kato et al [43] reported that SARS-CoV-2 induced the production of mucin (MUC)5AC and MUC5B throughout all airway regions via epidermal growth factor receptor/interleukin-1 receptor signaling activation in patients with COVID-19 [43]. This overproduction of airway mucin may increase the viscosity of the sputum, rendering it difficult to cough up. In addition, respiratory viruses infecting the human airway would impair mucociliary clearance, activate transient receptor potential vanilloid 1, induce the hypertrophy and metaplasia of goblet cells in the airway epithelium and the consequent enlargement of bronchial submucosal glands, leading to excessive fluid secretion and mucus plugs formation [42,44]. Intriguingly, we also observed that many individuals, especially the female participants, coughed up yellow or purulent sputum, suggesting coexistence of a bacterial infection. A previous study also suggested female sex as a susceptibility factor for bacterial infection [45]. However, it remains unclear whether these mechanisms play a pivotal role in mucus hypersecretion during Omicron infection.

Over 91% of individuals in our study had received a vaccine against SARS-CoV-2; however, we did not find a protective effect of the vaccine toward alleviating cough and mucus hypersecretion. The inactivated vaccines against SARS-CoV-2 were used in China; however, less than 15% of individuals had received a vaccine <6 months before the latest SARS-CoV-2 wave. Malik et al [46] demonstrated that the neutralizing antibody titer declines to near or below the seropositive threshold of a protective effect 6 months after vaccination with the inactivated vaccine. In addition, SARS-CoV-2 has been constantly mutating and most of the mutations are associated with the spike protein, a vital target for the action of vaccines [46]. Although a booster dose of the inactivated vaccine was associated with a lower risk of Omicron infection [47], compared with the ancestor strain, the production of neutralizing antibodies against Omicron was considerably impaired [48,49]. Another possible effect of the vaccine is the risk of antibody-dependent enhancement. According to in vitro studies, antibody-dependent enhancement might occur in COVID-19 infection [50,51], but current evidence indicates that this is an unlikely side effect induced by the vaccine [52,53].

There are some limitations of our study. First, all participants were nonhospitalized individuals with COVID-19, which may not fully reflect the cough profiles of severe cases of SARS-CoV-2 infection during the Omicron outbreak. Second, the data were obtained via online survey. Participants with low literacy or those of older age may have been neglected given the difficulties in filling out the survey and/or using a smartphone. In addition, clinical features and medical history were assessed by questionnaires, which may be prone to recall bias. However, most of the participants had been infected with SARS-CoV-2 within 1 month prior to completing the survey and many participants were still in the acute phase of COVID-19 at the time of the survey; hence, the impact of recall bias should be small. Lastly, we are following up with these respondents as we have only analyzed cross-sectional data; since many of the participants were still in acute phase of Omicron infection, the prevalence of persistent cough should be surveyed further.

In conclusion, over 90% of nonhospitalized individuals infected with the Omicron variant of SARS-CoV-2 presented with cough as a major symptom, with approximately one-third of individuals having a severe or productive cough, commonly with white thick and yellow purulent sputum, which developed into a persistent cough in a portion of these individuals. Female sex, chronic cough, asthma, GERD, diabetes, smoking, and/or vaccination for COVID-19 were associated with severe cough, productive cough, and/or persistent cough. Further studies are warranted to investigate the mechanism, treatment, and long-term prognosis of cough associated with Omicron variant infection, which will provide insight into how to treat SARS-CoV-2–induced cough.

## Appendix

Multimedia Appendix 1 Survey on cough characteristics and prognosis of COVID-19: Questionnaire V1.0.

Multimedia Appendix 2 Number of questionnaires from 31 provinces of the Chinese mainland. A darker blue color indicates more questionnaires.

## Abbreviations

GERD: gastroesophageal reflux disease
MUC: mucin
VAS: visual analog scale

## Data Availability

The data sets used and/or analyzed during the current study are available from the corresponding author on reasonable request.
