# Supplementary material for: Profiles of Cough and Associated Risk Factors in Nonhospitalized Individuals With SARS-CoV-2 Omicron Variant Infection: Cross-Sectional Online Survey in China
Source: JMIR Public Health Surveill. 2024 Feb 5;10:e47453. doi: 10.2196/47453 (PMC10877488; doi:10.2196/47453)
Supplement: Multimedia Appendix 1 [file publichealth_v10i1e47453_app1.docx]

**Survey on cough characteristics and prognosis of COVID-19 V1.0**

Cough is one of the most common symptoms of COVID-19, which seriously affects the quality of life of patients. To clarify the clinical characteristics and prognosis of COVID-19-associated cough and provide more evidence for the management of COVID-19 patients with cough, we sincerely invite you to participate in this [Survey on cough characteristics and prognosis of COVID-19 V1.0]. This questionnaire will take you 5 minutes to fill in. Thank you for your participation!

We promise that all information is only used for scientific investigation, and your personal information will not be disseminated or disclosed in any form.

Thanks again for your cooperation! Wish you a speedy recovery!

Chinese Cough Alliance

National Center for Respiratory Medicine

State Key Laboratory of Respiratory Diseases

2022/12/31

1. Your gender：[Single-choice] *

- Male □ Female

1. Your age：[Fill in the blank] *

_________________________________

1. Your telephone number: [Optional, fill in the blank]

_________________________________

1. Date of onset of COVID-19 symptoms [Fill in the blank] *

_________________________________

1. Date of the first positive result of COVID-19 antigen/nucleic acid [Fill in the blank]

_________________________________

1. Did you go to the doctor due to COVID-19? [Single-choice] *

□ Yes. I went to the clinic (or urgent care).

□ Yes, I was hospitalized.

□ No, I didn’t go to the doctor.

1. Have you experienced any of the following symptoms since infection of COVID-19? [multiple choice] *

□ Fever

□ Cough

□ Fatigue

□ Chest tightness

□ Nasal congestion

□ Runny nose

□ Postnasal drip

*Mucus dripping from the back of your nose to the throat*

□ Sore throat

□ Myalgia/ Arthralgia

□ Headache/ Dizziness

□ Diarrhoea

□ Conjunctivitis

□ Hyposmia/ Hypogeusia

□ Other symptoms not mentioned above_________________

1. Have you experienced any of the following severe symptoms during COVID-19 [multiple choice] *

□ High fever (≥39.1℃)

□ Severe sore throat (Sense of swallowing blade)

□ Severe nasal congestion (Sense of cement sealing nose)

□ Severe bone pain/ Myalgia

□ Severe headache

□ Shortness of breath / Dyspnoea

□ None of the above symptoms

1. How many days did it take from the onset of COVID-19to the presence of cough? [Fill in the blank] *

_________________________________

*Based on the option 2 of question 7*

1. Did you have sputum? [Single-choice] *

□ Yes, there is abundant sputum.

□ Yes, there is a little sputum.

□ No sputum.

*Based on the option 2 of question 7*

1. The color and characteristic of sputum [Single-choice] *

□ White and thick viscous sputum

□ White and watery sputum

□ Yellow and purulent sputum

□ Mainly white sputum and a little yellow sputum

□ White sputum at the beginning, then yellow sputum

□ Yellow sputum at the beginning, then white sputum

□ Blood-stained sputum

*Based on the option 1 or 2 of question 10*

1. Duration of COVID-19-associated cough (days, counted from the onset of cough symptoms) [Fill in the blank]

_________________________________

*Based on the option 2 of question 7*

1. Your main coughing time [Single-choice] *

□ Mainly daytime

□ Mainly Evening (before falling asleep)

□ Mainly nighttime (after falling asleep)

□ Cough both day and night

*Based on the option 2 of question 7*

1. Please rate the severity of your cough during the COVID-19 (0-100 points). The higher the score, the more serious you think the cough is. [Fill in the blank] *

_________________________________

*Based on the option 2 of question 7*

1. Is COVID-19-associated cough more severe than your previous cough after a common cold? [Single-choice] *

□ Yes, I have never cough after a common cold.

□ Yes, COVID-19-associated cough is more severe.

□ No, there is no difference between COVID-19-associated cough and previous cough after a common cold

□ No, COVID-19-associated cough is less severe than previous cough after a common cold.

*Based on the option 2 of question 7*

1. How was the result of chest CT/chest X-ray examination during/after COVID-19? [Single-choice] *

□ Chest CT/X-ray showed pneumonia

□ Chest CT/X-ray did not show pneumonia

□ Chest CT/X-ray was performed but the I couldn’t judge whether I had pneumonia

□ Chest CT/X-ray was not performed

1. Have you taken any of the following medicines during COVID-19? [multiple choice] *

□ Ibuprofen/acetaminophen/diclofenac or other antipyretics

□ Penicillin/cephalosporins/quinolones or other antibiotics (oral/intravenous infusion)

□ Lopinavir/ritonavir/ribavirin or other antiviral drugs

□Dextromethorphan/compound methoxyphenamine/loratadine/cough syrup or other antitussive

□ Naphazoline/Oxymetazoline Nasal Drops or other nasal medications

□ Traditional Chinese Medicine/Chinese Proprietary Medicine

□ Taking no medication

1. Have you ever had any of the following diseases? [multiple choice] *

□ Chronic obstructive pulmonary disease

□ Bronchial asthma

□ Chronic cough

*Continuous cough for more than 8 weeks*

□ Chronic rhinitis/sinusitis

□ Interstitial lung disease

□ Hypertension

□ Diabetes

□ Coronary heart disease

□ Cerebrovascular disease

□ Chronic gastrointestinal disease/gastroesophageal reflux disease

□ Malignant tumor

□ Chronic kidney disease

□ Chronic urticaria/allergic skin disease

□ No above diseases

1. Do you have a history of smoking? [Single-choice] *

□ Yes, currently smoking (or quit smoking no more than half a year)

□ Yes, have quit smoking for more than half a year

□ No, passive smoking (secondhand smoke)

□ No, never smoke

1. Have you been vaccinated against COVID-19? [Single-choice] *

□ Yes, three stitches has been completed

□ Yes, two stitches has been completed

□ Yes, one stitch has been completed

□ No, vaccination was not performed

1. Date of the last vaccination (according to the Health Code) [Fill in the blank] *

_________________________________

*Based on the option 1, 2, 3 of question 20*
