# Supplementary figures and images for: Profiles of Cough and Associated Risk Factors in Nonhospitalized Individuals With SARS-CoV-2 Omicron Variant Infection: Cross-Sectional Online Survey in China
Source: JMIR Public Health Surveill. 2024 Feb 5;10:e47453. doi: 10.2196/47453 (PMC10877488; doi:10.2196/47453)

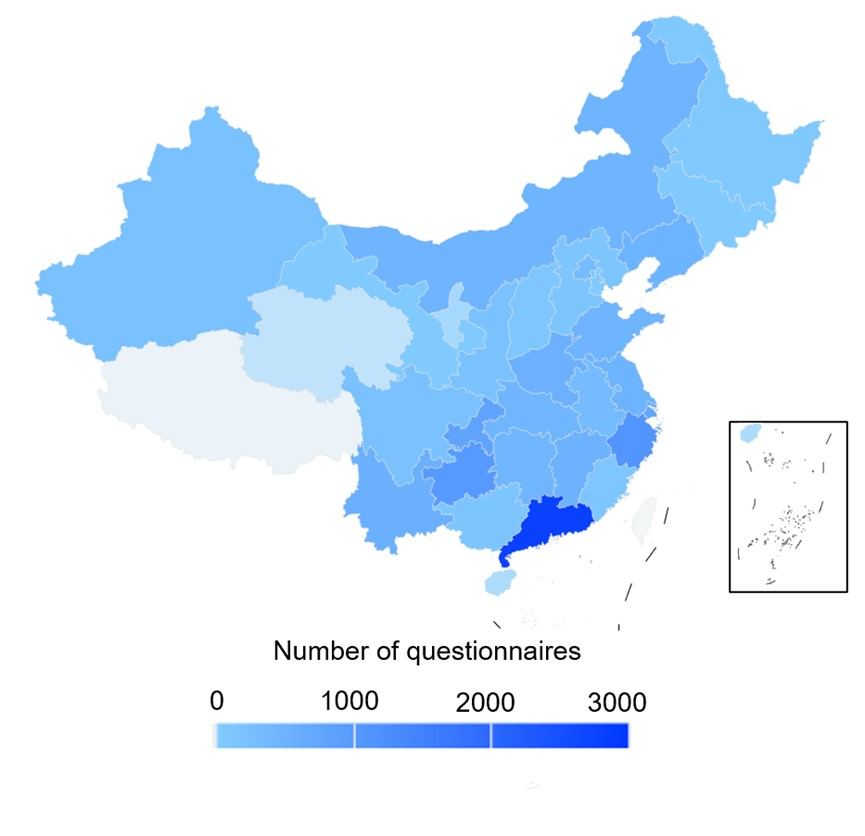

Supplement: Multimedia Appendix 2 [file publichealth_v10i1e47453_app2.png]
